# A Rare Case of Symblepharon

**Published:** 1890-05-03

**Authors:** 


### A RARE CASE OF SYMBLEPHARON.

Dr. P. Fischer, of Ueberlingen, reports a curious case in a woman aged 32. Complete cohesion had taken place between the conjunctivæ of the ball and both upper and lower lids, as the result of prolonged inflammation of a suppurative character, which made it impossible either to open or to quite close the eyes. Dr. Fischer having dissected the lids from the balls, replaced the lost conjunctiva by a portion of mucous membrane from another region of the patient's person, in one eye with complete success, but in the other a small gap remained, which was afterwards made good with mucous membrane from a rabbit's lip.

